# Vulnerability Assessment of Sensor Systems

**DOI:** 10.3390/s19112518

**Published:** 2019-06-01

**Authors:** Andrzej Bialas

**Affiliations:** ŁUKASIEWICZ Research Network—Institute of Innovative Technologies EMAG, 40-189 Katowice, Leopolda 31, Poland; Andrzej.Bialas@ibemag.pl; Tel.: +48-32-2007-700; Fax: +48-32-2007-701

**Keywords:** intelligent sensor, IT security evaluation, security assurance, Common Criteria, vulnerability assessment

## Abstract

There are more and more applications of sensors in today’s world. Moreover, sensor systems are getting more complex and they are used for many high-risk security-critical purposes. Security assurance is a key issue for sensors and for other information technology (IT) products. Still, sensor security facilities and methodologies are relatively poor compared to other IT products. That is why a methodical approach to the sensor IT security is needed, i.e., risk management, implementation of countermeasures, vulnerability removal, and security evaluation and certification. The author proposes to apply the main security assurance methodology specified in ISO/IEC 15408 Common Criteria to solve specific security problems of sensors. A new Common Criteria compliant method is developed which specifies the vulnerability assessment process and related data in a structured way. The input/output data of the introduced elementary evaluation processes are modeled as ontology classes to work out knowledge bases. The validation shows that sensor-specific knowledge can be acquired during the vulnerability assessment process and then placed in knowledge bases and used. The method can be applied in different IT products, especially those with few certifications, such as sensors. The presented methodology will be implemented in a software tool in the future.

## 1. Introduction

This paper concerns the security assurance of intelligent sensors and sensor systems and is focused on the assessment and removal of their security weaknesses, called vulnerabilities. 

Sensors are electronic devices based on microcontrollers. They measure physical quantities and convert them into signals read by an observer or instrument. Some of them contain actuators. Intelligent sensors can process measured values and may be organized in sensor systems. They use network technologies for integration, i.e., the wireless technology.

Sensors are considered IT products and their vulnerabilities are related to the processes of producing, processing, storing, and transferring information. Security is understood as the protection of integrity, availability, and confidentiality of information. The integrity of sensor systems is crucial as well as the continuity of services they provide. The security issue is very important for sensor systems due to following reasons.

1. Sensors are integrated with the use of network technologies, mainly wireless technologies, and make up complex IT systems. There are numerous sensor-specific threats and vulnerabilities. The risks related to these threats/vulnerabilities should be mitigated by using sensor-specific security measures. Additionally, sensor systems are connected to business, internet, and other systems. The threats diffusing from this upper layer and exploiting sensor vulnerabilities can penetrate sensor systems as well. Therefore, their impact should be mitigated too.
2. Please note that sensors, as edge devices, often work on the first frontier, having direct and immediate influence on human lives, the ecological environment, industrial installations, and transport-, energy-, and security monitoring systems, etc. Any breach of sensor behaviour may impact any object in its environment.
3. Sensor resources (computation, memory, power) and security facilities are relatively poor compared to other IT systems, but the risk is similar. Some threats are sensor-specific and some are the same as those of IT systems.
4. Using sensor vulnerabilities and other weaknesses, an intruder may get access to the entire system. Sensors cannot be the weakest links of complex systems.

The issue is how to enhance the security of sensors recognized as specific IT products. A possible way is to adapt known, proven methods to this specific domain. The question is which methods can be used or adapted and what the range of this adaptation could be. 

Generally, sensors are used in many high-risk security-critical environments where valuable assets exist and/or important services are provided. The risk is mitigated by countermeasures, which stand up against threats, or by removing vulnerabilities. The question is whether the countermeasure will act properly when the threat occurs and really will mitigate the risk in this critical situation. Basically, not all countermeasures can be applied, only the effective ones that guarantee security assurance. 

The assurance of an IT product means that the product meets its security objectives, i.e., the product-implemented security measures will be able to counter threats when they occur. The IT security assurance concepts and relationships between them are presented in the international standard [1]. Different technical mechanisms or methods applied to the IT systems can raise their assurance. With respect to sensors, examples of such mechanisms can be: a dedicated routing protocol for wireless sensor networks, as discussed in two papers [2,3], or reliable and fault-tolerant initial data storage, as presented in another paper [4]. Assurance can further grow when such mechanisms are well implemented, thoroughly analyzed, and tested by independent teams according to the defined schemas and methods. These activities are supported by the basic security assurance methodology, which is specified in the ISO/IEC 15408 Common Criteria (CC) standard [5]. This methodology provides confidence that countermeasures applied in the IT product (hardware, firmware, software, system) are relevant, sufficient, and correct, and will minimize risk in the operational environment. This is the foundation of the customers’ confidence in the IT product. Relations between security and evaluation concepts: asset–threat–vulnerability–risk–countermeasure–evaluation–certifications–security assurance are discussed in Part 1/Section 7 of [5]. The assurance is based on the rigor applied during the development and manufacturing processes, independent third-party evaluation, and operation and maintenance according to the obtained certificate. IT products which are developed and evaluated in conformance with these requirements can be applied in higher risk environments. According to the Common Criteria methodology, the assurance is measurable using EALs (evaluation assurance levels) in the range of EAL1 to EAL7. This methodology has been broadly used for years. More than 2000 certification processes have been completed so far and the certificates have been granted to varied products or systems [6].

The Common Criteria methodology is defined on the general level and for this reason, it can be applied to any kind of IT product, but during the IT product development and evaluation processes, the distinctive characteristics and facilities of the IT product should be considered. This is performed by the identification of the IT product-related protected assets, vulnerabilities and threats, characteristics of the IT product operation environment, applications, risk, roles, etc. All this identified information is used to refine or supplement the Common Criteria development and evaluation processes, especially their input/output data. The methodology presented in the paper can be considered an example of the CC extension and refinement.

With respect to the CC methodology, intelligent sensors can be considered relatively new and specific IT products. They have hardware (integrated circuits, electronic modules), firmware, network elements, and software (databases, applications) components and they co-operate with other IT systems. The risks related to the use of sensors in a broad range of business, industrial, health, and social applications should be taken into consideration. The mentioned heterogeneity, restricted power, processing, and communication resources, simplified security mechanisms, and specific threats and vulnerabilities strongly influence sensors’ security development and evaluation. 

Please note that the number of certified sensors or sensor systems is currently low, though the demand for certified IT products, including sensors, IoT/IIoT (Internet of Things/Industrial Internet of Things) and other compact devices has been increasing recently. The Common Criteria application in sensors and sensor systems development are presented in [7,8,9,10,11,12,13,14,15] and will be discussed in the Section 2.3.1. Sensor products are similar to hardware security boxes distinguished by SOG-IS [16]. To meet this rising demand, the European cybersecurity certification schemes have been developed. The scheme integrates different evaluation/certification paths and assurance levels into one coherent scheme [17]. The research presented in the paper is consistent with these efforts. Sensor-specific vulnerabilities and attacks are discussed in numerous existing publications [18,19,20,21], which can be used to form knowledge bases. The security evaluation and vulnerability assessment, especially in the Common Criteria domain, is presented in [22,23,24,25,26,27,28], which will be discussed in Section 2.3.2.

The purpose of the work presented in the paper is to assess whether the vulnerability assessment specified in the Common Criteria methodology can be directly applied to sensors or whether it requires adaptation, and if so, what the range of this adaptation should be. Please note that the vulnerability assessment is the key task of the security evaluators. 

The main paper contribution is the sensor system vulnerability assessment method (SVAM) developed as the result of the above-mentioned research. SVAM is based on the proven Common Criteria methodology and considers sensor-specific issues. SVAM refines and extends the general Common Criteria vulnerability assessment methodology by adapting it to the distinctive needs of sensors. SVAM defines vulnerability assessment activities and data provided and produced by these activities. It can be applied in the Common Criteria evaluation labs (ITSEFs—IT Security Evaluation Facilities) or, alternatively, by sensor system developers to remove vulnerabilities on early stages of the sensors development. SVAM is compliant with the CEM [29] and the related data models. SVAM embraces the basic activities of the vulnerability assessment:

- identification of the sensor-specific security issues orientating the vulnerability assessment,
- identification of publicly available information about vulnerabilities,
- performing penetration tests during the vulnerability assessment,
- elimination, minimizing, or monitoring vulnerabilities which may be introduced by the developers to their solutions.

The proposed SVAM is an example of knowledge engineering applications in the Common Criteria domain. There are many publications and solutions in this field [30,31,32]. Another example, which does not concern sensors security, is a publication on the maintenance of industrial machines based on sensors and ontology [33]. They will be discussed in Section 2.3.3.

Section 2 presents the paper’s background, i.e., a short description of the Common Criteria standard (Section 2.1), the Common Criteria approach to the vulnerability assessment process (Section 2.2), and the current state of research domain (Section 2.3). 

The main part of the paper (Section 3) presents the developed vulnerability assessment method. Section 4 includes the method validation on a sensor example and Section 5 is a discussion of the obtained results. The final section concludes the paper.

## 2. Background and Related Work

This section presents two issues:

- basic terms related to the Common Criteria methodology, especially the vulnerability assessment approach, which are the foundation of the presented SVAM method;
- the state of the art related to the paper’s content, including all research threads in the paper: Common Criteria applications in sensors, vulnerability assessment, and knowledge engineering applications in security.

### 2.1. Common Criteria Primer

There are three basic processes in the CC [5] methodology:

- the IT security development process of an IT product called Target of Evaluation (TOE); after different security analyses, a document is prepared, called Security Target (ST); the ST embraces the security problem definition (SPD) and its solution by specifying security objectives (SO), security requirements, and functions; security functional requirements (SFRs), derived from SOs, describe how security measures should work in the operational environment; EAL-related security assurance requirements (SARs) determine how much assurance one can have in the IT product; the ST comprises TOE security functions (TSFs) which meet SFRs; the TSFs are implemented on the claimed EAL;
- the TOE development process (according to the SAR components embraced by the claimed EAL) concerns the IT product (TOE) and its documentation;
- the security evaluation process conducted according to CEM in an independent laboratory (ITSEF), supervised by the given national certification body [6].

The results of the first two processes are called evaluation evidences. They are transferred together with the TOE to the ITSEF.

Part 2 of the CC standard [5] comprises components to express security functional requirements (SFRs) used to model the IT product’s security behavior. Part 3 of the CC standard [5] includes components to express security assurance requirements (SARs) of this product. The components described in both parts are grouped by families, which, in turn, are grouped by classes, e.g.,:

- the “FDP” functional class concerns the user data protection, its family “FDP_ACC” concerns the access control policy, and the family component “FDP_ACC.2” expresses the issue of “Complete access control”;
- the assurance class “ATE” concerns tests, its family “ATE_DPT” (depth of testing) deals with the level of details to which the security functions are tested by the developer; component “ATE_DPT.1 Testing: basic design” provides assurance that the TSF subsystems behave and interact as described in the TOE design and the security architecture description.

Each SAR component has evaluation evidence assigned, provided by the developer and assessed according to the common evaluation methodology (CEM) [29]. The CC “SAR components” correspond to the CEM “subactivities”, which are divided into evaluation actions. The evaluator investigates the evidence and/or the TOE behavior and assigns verdicts (pass/fail/inconclusive) to the evaluation actions.

The IT product assurance rises when more effort is devoted to its development and evaluation. This effort depends on the TOE scope (less or more portion of the IT product is evaluated), depth (fewer or more design and implementation details considered), and rigor (a more or less structured and formal approach is applied).

The Common Criteria methodology (CC, CEM) is described in publications worldwide [34,35] and in the author’s publications [7,36,37,38].

### 2.2. Vulnerability Assessment According to the Common Criteria Methodology

Generally, a vulnerability is understood as a weakness of the IT product (TOE) that can be used to violate the security requirements (SFRs) in the operational environment. Vulnerabilities are caused by failures in the security requirements identification, in the TOE development, e.g., possibility of defeating self-protection through tampering, or in operation, e.g., insecure configuration of the TOE. Threat agents may intentionally exploit or unintentionally trigger a vulnerability, causing security breaches in IT business applications. For these reasons, vulnerabilities should be removed, neutralized, minimized with respect to their consequences, and monitored.

The vulnerability assessment is the key activity of the IT security evaluation process. It is based on the requirements (CC components) belonging to the vulnerability assessment assurance class (AVA), which contains one family called AVA_VAN (vulnerability analysis) including five assurance components with the rising and cumulated rigor, (part 3 of the Common Criteria standard [5]):

- AVA_VAN.1 Vulnerability survey (applied for EAL1),
- AVA_VAN.2 Vulnerability analysis (applied for EAL2 and EAL3),
- AVA_VAN.3 Focused vulnerability analysis (applied for EAL4),
- AVA_VAN.4 Methodical vulnerability analysis (applied for EAL5),
- AVA_VAN.5 Advanced methodical vulnerability analysis (applied for EAL6 and EAL7).

Each AVA_VAN component, like each other CC assurance component, has three kinds of elements:

- D-element, expressing what should be delivered by the developer as the evidence, e.g., “AVA_VAN.3.1D Developer shall provide the TOE for testing”;
- C-element, i.e., contents and presentation of this evidence item, e.g., “AVA_VAN.3.1C The TOE shall be suitable for testing”;
- E-evaluator’s action elements, denoted, e.g., as AVA_VAN.3.1E, express how this evidence item shall be checked by the evaluator; for each subactivity (a component) one or more evaluator’s actions elements exist (to be discussed later); to each action, a verdict is assigned; each action has several “work units” structuring properly and refining the evaluator’s activity.

The objective of AVA_VAN is to determine whether the identified potential vulnerabilities could allow the attacker to violate the SFRs. Using the evaluation evidence and public information, the evaluator performs the vulnerability analysis according to CEM [29] and carries out penetration tests. During the vulnerability assessment a wide range of assessment methodologies, unspecific to the kind of an attack scenario, can be applied. The CC vulnerability assessment is relatively specific compared to other vulnerability assessment services offered on the market to IT systems used in organizations:

- it is better defined on a general level;
- it should provide repeatability of assessments performed in different ITSEFs;
- it considers “a type”, i.e., a group of IT products, not a concrete system applied in an organization;
- it considers the assumed operational environment of an IT product;
- it embraces multidirectional evaluation activities: examination of generally known vulnerabilities described in public information sources, analysis of evaluation evidences to find vulnerabilities introduced by developers, less or more formal vulnerability analyses (including the flaw hypotheses), assessment of resistance to a given attack potential, and experimental confirmation of the analysis results by penetration tests;
- it is multilayered—the analytical rigor depends on the EAL and is specified by AVA_VAN.x assurance components.

### 2.3. Current State of the Research Field

The review is focused on three basic issues:

- Common Criteria application in sensor and sensor system development;
- Security evaluation and vulnerability assessment, especially in the Common Criteria domain;
- Knowledge engineering applications in the Common Criteria domain and cyber security knowledge bases.

#### 2.3.1. Common Criteria Application in Sensors and Sensor Systems Development

The paper [7] contains a survey of the Common Criteria applications in the sensor systems domain. The most relevant of them concern:

- general sensor network security aspects;
- healthcare systems;
- aircraft health monitoring systems;
- safety-critical assets distribution systems;
- transport systems, including motion sensors of digital tachographs;
- products related to SCADA (supervisory control and data acquisition);
- specialized firewalls used in control and automation systems, co-operating with sensors networks.

A previous paper [8] considered the analysis of wireless sensor network (WSN) security based on the regulations intended for wireless communication devices. It discusses WSN attacks, security measures countering these attacks, and provides a security evaluation and classification methodology for WSN protocols (a communication stack). The Common Criteria methodology is proposed as the validation methodology of security countermeasures in WSN systems. The countermeasures’ sufficiency (SFRs-based) and correctness (SARs-based) are evaluated. The countermeasures, identified as security objectives, are expressed with the use of the selected SFRs related to:

- nonrepudiation of the origin (FCO_NRO) and source (FCO_NRR)—source and destination identifier in each message,
- user identification (FIA_UID)—introducing “membership control-bus guardian”,
- different mechanisms related to the “FDP user data protection” class.

The threat/attacks and countermeasures reference lists [8] can be used as information sources about WSN attacks and vulnerabilities in the SVAM method and to work out knowledge repositories for evaluators. 

A chapter in [9] is focused on the security and privacy issues in healthcare systems, including sensor and wireless sensor networks. Security and privacy threats are mapped to the commonly used countermeasures. The countermeasures are discussed with respect to the limits of the sensor networks, as it is difficult to apply advanced cryptographic techniques to them. The chapter remarks that assurance relies on engineering practices, development processes, operational issues, and can be evaluated according to the Common Criteria methodology. 

Below are different examples of Common Criteria applications (security analysis of a system, identifying threats, deriving security objectives, requirements, and security functions) in the sensor systems domain concerning e-Enabled aircrafts [10,11]:

- Electronic distribution of software (EDS),
- Airplane assets distribution system (AADS),
- Air health monitoring and management system (AHMMS),
- Air traffic control (ATC).

The automotive industry uses similar communication channels to load assets, i.e., software or contents, to different embedded electronic control units built into vehicles [12].

The digital tachograph system [13,14,15] consists of a vehicle unit, motion sensor, and a smart card used to log in to the unit. The Common Criteria standard is used to evaluate the security of tachograph systems.

The review of these few examples concludes that the Common Criteria methodology is rarely directly used in the sensor and sensor system certification processes. Among many certified IT products (more than 2000) and registered protection profiles (more than 300), only a few are directly related to sensors and sensor systems, i.e., motion sensors for digital tachographs and industrial control systems that include sensors. 

Apart from these certified IT products, the Common Criteria methodology is used:

- for noncommercial, e.g., military purposes, but they are not well documented,
- as a supporting methodology to solve design problems (without certification) [8,9].

#### 2.3.2. Security Evaluation and Vulnerability Assessment, Especially in the Common Criteria Domain

The Common Evaluation Methodology (CEM) [29] is complex, but still provides some general solutions that should be suitable to any kind of IT product (integrated circuits, firmware, software, hardware devices, complex systems). For these reasons, different product-specific guidelines have been developed. Some of them concern vulnerability assessment, which is a significant part of CEM and a very important activity of the CC evaluators.

The standard [22] provides an extension of the basic CEM methodology to evaluate software products. It shows how to use public databases of vulnerabilities and attacks, but only for software products.

The guide [23] supplements and refines the CEM methodology from the developer’s point of view. 

The presentations delivered at the International Common Criteria Conferences (ICCC) concern different aspects of the vulnerability assessment raised during IT security evaluations in labs. For example:

- [24] proposes to mitigate CEM drawbacks, i.e., “a very generic vulnerability classification and poorly defined methodology” by applying the attack patterns from CAPEC [25], better aligning to SAR classes/families, such as AGD—misuse, ALC—delivered vulnerabilities, ATE—malfunction, ADV_ARC, ADV_TDS, ASE_SPD—attack path;
- [26] concerns a new approach to the patch management after certification in the context of vulnerability and risk management;
- [27] concerns test automation; penetration tests are part of the vulnerability assessment.

Another report [28] specifies methods and tools for vulnerability analyses designed for specific IT products or systems, such as network scanners, host scanners, web application scanners, multilevel scanners, penetration test tools, vulnerability scan consolidators, etc. Some methods and tools can be used in penetration testing, as proposed below.

The CEM methodology can be refined with respect to the given group of IT products.

#### 2.3.3. Knowledge Engineering Applications in the Common Criteria Domain and Cybersecurity Knowledge Bases

A thorough literature survey on “security assessment ontologies” made the following conclusions [30]:

- “Most works on security ontologies aim to describe the Information Security domain (more generic), or other specific subdomains of security, but not specifically the Security Assessment domain”;
- there is “a lack of ontologies that consider the relation of Information Security and Software Assessment fields of research”;
- there is “a lack of works that address the research issues: Reusing Knowledge; Automating Processes; Increasing Coverage of Assessment; Identifying Vulnerabilities; Measuring Security; Assessing, Verifying or Testing the Security”.

Another paper [31] conducted a broad survey (more than 200 papers) of scientific publications devoted to ontologies for privacy requirements engineering. Only two papers concerning Common Criteria were found. One of them [32] is focused on the ontological representation of the Common Criteria components. On this basis, a tool was developed to support the evaluator during the certification process, i.e., to plan the evaluation process, review relevant documents, or make reports. The aim of this work is to decrease the time and costs of security evaluation. This ontology is general and embraces all CC assurance components but it does not express details required for work units, especially for the vulnerability assessment, e.g., public sources of information about vulnerabilities, attacks, areas of concerns, and attack potential.

Another paper [39] considered the development of CC ontology and an ontology-based tool supporting CC knowledge query, mark-up, review, and report functions to better understand CC and enhance the effectiveness of CC certification. This ontology does not embrace CEM activities, especially of the AVA assurance class.

Another paper [40] presented core ontology concepts for security assessment. This approach is general and does not comply with Common Criteria. These concepts may be used to develop the vulnerability assessment ontology.

Another paper [41] discussed cyber security ontology related to common sources of information of IT products, assets, attacks, threats, and, first and foremost, vulnerabilities, including [25,42,43,44,45,46,47] and others. These commonly used knowledge bases are for cybersecurity management purposes. This ontology can also be useful to organize access to these sources during the Common Criteria vulnerability assessment process, which supports searching these sources for potential vulnerabilities for the evaluated IT products. 

The NIST document [48] presents a vulnerability ontology specification. The ontology includes the minimum information needed to properly inform the vulnerability management process and to facilitate the sharing of vulnerability information. It is used by vulnerability data feeds and for vulnerability scoring [49]. This approach is used in the method discussed in this paper.

Another previous work [50] discussed a high-level ontology involving different sources of cyber security information. It is focused on the needs of security operation centres (SOC). The solved problem is very similar to the CC vulnerability assessment, although the CC process is focused on the vulnerability of a specific IT product, e.g., a sensor.

There is neither a method nor a tool, especially ontology-based, to support the vulnerability assessment activities according to the CC standard, e.g., the search for public information sources for vulnerabilities, evidence-based vulnerabilities, structured vulnerability analyses, and penetration test management. This was a motivation for the author to support the vulnerability assessment process of IT products, especially specific ones, such as sensors. 

## 3. Sensor System Vulnerability Assessment Method (SVAM)

### 3.1. The Problem Origin

The Common Criteria IT security evaluation process embraces the whole TOE evidence material according to the claimed EAL. The most important part of these evidences related to the vulnerability assessment is discussed herein. This assessment is closely related to the AVA_VAN subactivities [29], here from AVA_VAN.1 to AVA_VAN.4, because the guidance for AVA_VAN.5 is not available (“There is no general guidance; the scheme should be consulted for guidance on this subactivity” [29], pp. 346).

The sensor system vulnerability assessment method (SVAM) was developed based on a detailed analysis of the AVA_VAN subactivities (components) to identify: operations performed by evaluators working in ITSEFs, information needed for these operations, and results of these operations used to compose final evaluation verdicts. The overview is focused on the issue of how to support evaluators in AVA_VAN works, i.e., how to facilitate their access to relevant information, how to work out evaluation decisions more easily, how to express this information more precisely (formally), and how to help the evaluators in analyses, calculations, and penetration testing.

All evaluator actions (i.e., E-elements) can be grouped thematically, but their rigor and details expressed by their work units differ. 

There are four AVA_VAN evaluation issues (implied by E-elements) common to all subactivities:

1. AVA_VAN.1.1E, AVA_VAN.2.1E, AVA_VAN.3.1E, AVA_VAN.4.1E elements concern the issue: how to check whether the delivered TOE (e.g., a sensor system) is well prepared for the analyses and penetration tests.

The actions concern the TOE configuration, compliance of the operational and test environments, test equipment, installation, and initialization (e.g., reaching “a known state after the power on”). 

These actions are similar for all IT products and are based on general routines related to the evaluated product and its evidences—no specific research issues focused on supporting the evaluator have been identified by the author.

2. AVA_VAN.1.2E, AVA_VAN.2.2E, AVA_VAN.3.2E, AVA_VAN.4.2E elements concern how to examine publicly available information sources to identify potential vulnerabilities.

There are many sources of publicly available, security-related information presenting an extremely broad range of issues, e.g., mailing lists, security forums, publications, etc. They have different features, contents, and quality. The search is time-consuming and the search results’ quality is questionable because certain relevant vulnerabilities may be omitted. This process requires better orientation in terms of the TOE’s specific features (here, sensor-specific) and better structure and organization. Currently no guidelines are available on this matter. The questions are: “How to identify representative and plausible sources?” and “How to identify the potential vulnerability relevant to the evaluated TOE?”. 

The research problem is to develop an ontology-based method allowing one to:

- identify security properties of the evaluated IT product relevant to the vulnerability assessment;
- identify a potential vulnerability process using these properties.

3. AVA_VAN.2.3E, AVA_VAN.3.3E, AVA_VAN.4.3E elements concern how to perform a less or more rigorous vulnerability analysis (AVA_VAN.2.3E—independent, AVA_VAN.3.3E—focused, AVA_VAN.4.3E—methodical).

The vulnerability analysis is based on the search for evidence—security target (ASE), guidance documentation (AGD), functional specification (ADV_FSP), TOE design (ADV_TDS), security architecture (ADV_ARC), and implementation (ADV_IMP)—to identify other potential vulnerabilities. This process is iterative, complex, and has to control a lot of diversified information. First, the hypothesized vulnerabilities are identified. Next, they are prioritized on the basis of the estimated probability that a potential vulnerability exists. Then, the attack potential required to exploit a vulnerability is considered. A prioritized list of potential vulnerabilities is used during penetration testing.

The research problem is to develop an ontology-based method allowing one to:

- identify vulnerabilities specified during the evaluation evidence analysis,
- assess whether they are applicable in the TOE operation environment,
- analyze their characteristics with respect to the possibility of bypassing, tampering, direct attacks, monitoring, misuse, and other threats provided by the evaluation authority,
- consider the attack potential required to exploit the given vulnerability.

4. AVA_VAN.1.3E, AVA_VAN.2.4E, AVA_VAN.3.4E, AVA_VAN.4.4E elements concern how to develop and conduct penetration tests based on the identified potential vulnerabilities to determine whether the TOE is resistant to attacks performed by an attacker possessing appropriate attack potential (basic, enhanced basic, moderate, high).

The evaluator devises penetration tests based on potential vulnerabilities, produces penetration test documentation, conducts tests, reports, analyzes, and summarizes tests results. 

The research problem is to develop a tool supporting the management of the penetration tests, i.e., planning, documenting, supervising the equipment, test conducting, reporting, and summarizing. 

### 3.2. The Solution

Based on the analysis of the AVA_VAN subactivities (components), the sensor system vulnerability assessment method (SVAM) is proposed, which includes:

- elementary processes compliant with the AVA_VAN subactivities, which express the evaluators’ operations more precisely,
- models of input and output data of these elementary processes.

**Figure 1:**
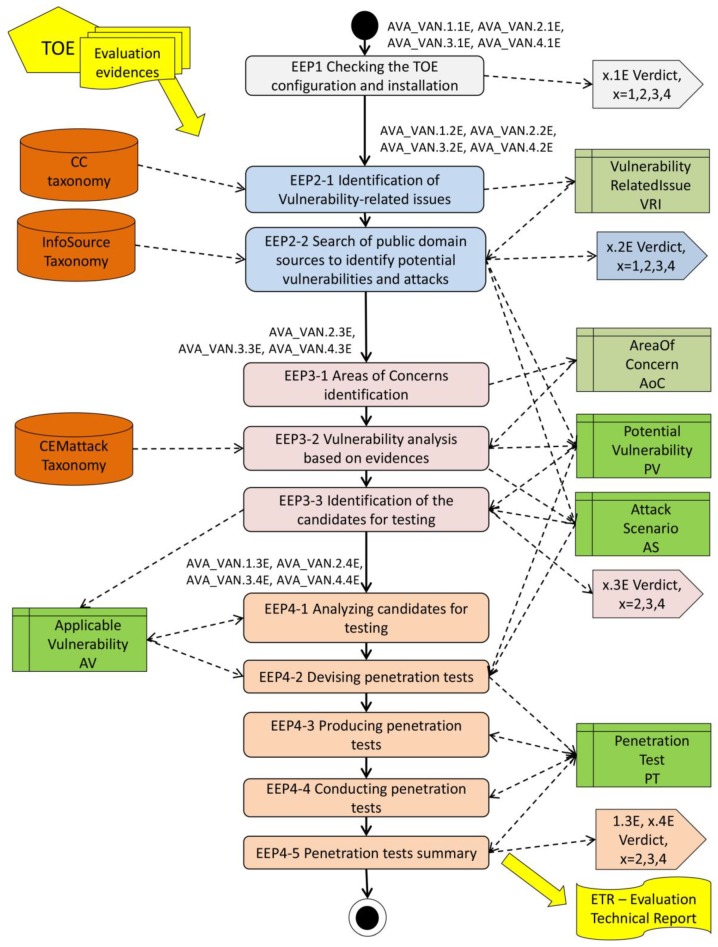
Elementary processes and input/output data of the sensor system vulnerability assessment method (SVAM).

Figure 1 presents elementary evaluation processes (EEPs) and related data. EEPs are grouped by E-elements. Please note that evaluation verdicts are represented by pentagons. AVA_VAN.1 (an exception) is focused only on vulnerabilities identified in external sources. Evaluation evidences are analyzed for other vulnerabilities too. 

#### 3.2.1. Elementary Evaluation Processes

The main AVA_VAN process was decomposed to the following elementary evaluation processes:

- For the AVA_VAN.1.1E, AVA_VAN.2.1E, AVA_VAN.3.1E, AVA_VAN.4.1E elements:
  — EEP1 Checking the TOE configuration and installation;
- For the AVA_VAN.1.2E, AVA_VAN.2.2E, AVA_VAN.3.2E, AVA_VAN.4.2E elements:
  — EEP2-1 Identification of vulnerability-related issues;
  — EEP2-2 Search of public domain sources to identify potential vulnerabilities and attacks;
- For the AVA_VAN.2.3E, AVA_VAN.3.3E, AVA_VAN.4.3E elements:
  — EEP3-1 Areas of concerns identification;
  — EEP3-2 Vulnerability analysis based on evidences;
  — EEP3-3 Identification of the candidates for testing;
- For the AVA_VAN.1.3E, AVA_VAN.2.4E, AVA_VAN.3.4E, AVA_VAN.4.4E elements:
  — EEP4-1 Analyzing candidates for testing;
  — EEP4-2 Devising penetration tests;
  — EEP4-3 Producing penetration tests;
  — EEP4-4 Conducting penetration tests;
  — EEP4-5 Penetration tests summary.

The preliminary research results on EEPs have been discussed previously [51] and are expressed there in a pseudocode. The presented method can be applied for any kind of evaluated IT product, but it should be interpreted and refined for the given kind of product. 

The TOE and all its evaluation evidences are used as the AVA_VAN process input. The result of the AVA_VAN evaluation is placed in the evaluation technical report (ETR) as the process output. 

EEP1 checks if the TOE is properly configured and installed, i.e., is ready for evaluation experiments and testing. 

EEP2-1 identifies the TOE-specific characteristics to orientate the potential vulnerability search in public information assets. The search is performed in EEP2-2. 

EEP3-n concerns searching for vulnerabilities by the analysis of evaluation evidences (omitted for the AVA_VAN.1 component) and involves the identification of areas of concern (vulnerability-prone parts of the TOE design), thus orientating the analysis. The analysis is performed in EEP3-2. EEP3-3 checks whether the given potential vulnerability is applicable to the TOE in its operational environment. Such vulnerabilities are subjects of further analyses and testing. 

EEP4-1 preselects the candidates for testing by a deeper analysis, e.g., it checks whether the TOE is resistant to the required attack potential (AP). For the preselected pairs, i.e., attack scenario–vulnerability, penetration tests are devised (EEP4-2), developed (EEP4-3), performed (EEP4-4), concluded, and finally reported in the ETR (evaluation technical report) in EEP4-5. As a result, exploitable and residual vulnerabilities are identified (exploitable, but only by attacks with potential higher than that considered for the claimed EAL).

#### 3.2.2. Modeling of Input and Output Data of Elementary Processes

During the vulnerability assessment, huge and diversified data assets (structured or unstructured) should be managed. Such data can be found in evaluation evidences provided by developers, in documents developed by the Common Criteria community around the world, in scientific and technical publications, in internet services, etc. Evaluators need preselected information of good quality to make evaluation decisions. The SVAM organizes these data around elementary evaluation processes. The data are identified and specified as knowledge bases developed on the ontology basis. Two groups of knowledge bases (KB) are distinguished:

- General-purpose KB containing commonly used knowledge, used for any evaluation project run in ITSEF, e.g., *CCtaxonomy*, *InfoSourceTaxonomy*, *CEMattackTaxonomy*, they are marked orange in Figure 1;
- Project-specific KB containing knowledge acquired and used in the given IT product evaluation (marked green in Figure 1), e.g., *PotentialVulnerability_PV*, *ApplicableVulnerability_AV.*

*CCtaxonomy* expresses the taxonomy used to classify all evaluated IT products and protection profiles [6]. It ensures compatibility with international products’ knowledge bases and allows one to identify a product similar to the evaluated one and, indirectly, find its potential vulnerabilities. 

*CEMattackTaxonomy* is an implementation of attack categories (and subcategories), such as bypassing, tampering, direct attacks, monitoring, and misuse, as proposed in CEM/B2.1 [29]. This KB includes all known and possible attacks (specified in the CC style: threat agent, exploited vulnerability, adverse action, impact).

*InfoSourceTaxonomy* represents all possible sources of information where potential TOE-related vulnerabilities can be found. These sources are diversified and external. 

*VulRelatIssue_VRI* is used to orientate the potential vulnerabilities search in the external information sources (EPP2-2). *AreaOfConcern_AOC* does the same but with respect to potential vulnerabilities hidden in the TOE evaluation evidences (EPP3-2). *VulRelatIssue_VRI* includes items of different categories represented by keywords, characterizing the TOE and its security. *AreaOfConcern_AOC* points out suspected areas of TOE design where vulnerabilities may occur, e.g., solutions too complicated or based on unproven technologies.

The *PotentialVulnerability_PV* knowledge base embraces identified potential vulnerabilities resulting from the search for external sources or analyses of the vulnerability-relevant items in evidences. Concurrently, possible attack scenarios exploiting potential vulnerabilities are identified and placed into the *AttackScenario_AS* knowledge base. Both these KBs are analyzed (EEP3-3) in terms of whether each potential vulnerability is relevant to the TOE’s operational environment. Those that are “applicable” are included in the *ApplicableVulnerability_AV* knowledge base, forming the basis for further analyses and penetration tests. Penetration tests, experimentally confirming if particular vulnerabilities are exploitable or not, are used to develop the ultimate AVA_VAN evaluation decision. The *PenetrationTest_PT* knowledge base includes tests and their results. 

#### 3.2.3. Software Support of the Vulnerability Assessment Methodology

The research on the structure of the AVA_VAN process and related data is aimed at the development of the software supporting evaluation works, which are generally complicated and require instant and good quality data to make decisions. The reusability of knowledge is also important because it may decrease the cost and time of the evaluation. 

A previous paper [51] considered AVA_VAN process structure. The structure data presented here is based on the developed ontological models related to AVA_VAN and is an extension of ontologies (CCO—Common Criteria ontology) previously developed by the author [52,53]. 

The paper is based on knowledge engineering principles and the Protégé v.5 tool developed at the Stanford Center for Biomedical Informatics Research [54,55]. The ontological models will be explained during their validation for sensor systems.

## 4. Validation of the Sensor System Vulnerability Assessment Method

A complex validation process requires a complete set of evaluation evidences at the evaluators’ disposal and is beyond the scope of this paper. Due to the information, cost, and time restrictions, the validation should be focused on the selected but representative operations performed by the evaluators.

The validation is based on the CEM requirements and on the documentation of the MEDIS sensor project—one of two sensors developed by the author’s organization which were used in the CCMODE validation. 

The validation is focused on:

- meeting the CC/CEM requirements related to the content and presentation of evidence and compliance with the work-units defined in CEM,
- the use of preselected information on attacks, vulnerabilities, information sources, penetration tests related to well-known, certified groups of IT products, such as firewalls, security boxes, etc., and information, which can be also applied to emerging groups of certified IT products, such as sensors,
- the use of sensor-relevant data included in the literature, security targets, and certification reports.

The aims of the validation are:

- to check the modelled AVA_VAN process with respect to the actions and data models of any IT product;
  — the validation conclusions will be used to develop the software tool supporting the evaluators;
  — expected result: confirm, reject, or modify the validated model before the software is developed on their basis;
  — consider the automation of the vulnerability assessment process by developing the software tool;
- to show how an IT product, such as a sensor system, is evaluated according to the Common Criteria with respect to vulnerabilities;
  — presentation of SVAM (sensor system vulnerability assessment method);
  — expected result: provide the method and knowledge for sensor developers and evaluators;
  — support the evaluators and developers of sensor systems in vulnerability assessment and removal.

The validation comprises different evaluation cases related to all evaluator action elements, as exemplified by ontological models. The following cases embrace all steps of the SVAM. 

### 4.1. Case 1: Checking If the Information Provided by the Sensor Developer Meets All Requirements For Content and Presentation of Evidence (AVA_VANx.1E, x = 1,2,3,4) 

Let us consider a hypothetical (i.e., not CC-certified) but representative sensor example, developed by the author’s organization [37], i.e., the methane early detection intelligent sensor (MEDIS), working within the broader mine monitoring system (MMS) controlling different safety and production parameters, e.g., physical parameters, chemical composition of air, as well as the condition and working parameters of ventilation equipment, machines, and process-line equipment. MMS is designed for mines with methane and coal-dust explosion hazards. 

Case 1 concerns the straightforward execution of “EEP1 Checking the TOE configuration and installation”:

1. Examination of whether the TOE test configuration is consistent with the configuration under evaluation as specified in the Security Target (ST) [29]. The evaluator checks whether the provided MEDIS sensor has the same configuration as specified in the TOE identifier of ST (here: MEDIS—methane early detection intelligent sensor, ver. 0.9 [37]) and in the ALC_CMC (configuration management) and ALC_CMS (configuration lists) evaluation evidences.
2. Examination of whether the TOE has been installed properly and is in a known state after the power on [29]. The evaluator performs the installation and preparation procedure of the MEDIS sensor specified in the AGD_PRE (preparative procedures) evidence document and checks the result.
3. The evaluator assigns the verdict (PASS/FAIL/INCONCLUSIVE) for AVA_VANx.1E and justifies it.

### 4.2. Case 2: Search of Public Domain Sources to Identify Potential Vulnerabilities in the TOE (AVA_VANx.2E, x = 1,2,3,4)

Case 2 is very complex and extensive because IT products may have diversified hardware- and software-specific vulnerabilities and public information sources are numerous and diversified. For this reason, it is divided into two issues:

- search planning—performed by “EEP2-1 Identification of Vulnerability-related issues”,
- search execution—performed by “EEP2-2 Search of public domain sources to identify potential vulnerabilities and attacks”.

EEP2-1 identifies keywords describing the TOE, e.g., the MEDIS sensor, as precisely as possible:

1. The evaluator reviews the TOE characteristics included in the Security Target (mainly its introduction), such as TOE type, description, and overview, and identifies adequate keywords, attributes, or statements for the future search for potential vulnerabilities. More details of the MEDIS example can be found in Section 4.1 [37].
2. The evaluator looks for differences and similarities to earlier evaluated products, reviewing a knowledge base in the CC portal [6], with the use of the general *CCtaxonomy*. These categories may also serve as keywords.
3. All keywords, attributes, or statements which best characterize the evaluated IT product are placed into the *VulRelatIssue_VRI* knowledge base.

The EEP2-1 input is:

- the security target of the evaluated product, especially “ST Introduction” with the above-mentioned TOE characteristics,
- the CC portal [6]—a knowledge base including the simplified ST and ETR of the evaluated products, grouped according to *CCtaxonomy*: access control devices and systems, biometric systems and devices, boundary protection devices and systems, data protection, databases, etc.

The EEP2-1 output is:

- the *VulRelatIssue_VRI* knowledge base (keywords, attributes, or statements precisely characterizing the TOE, defining the range of the planned searching).

**Figure 2:**
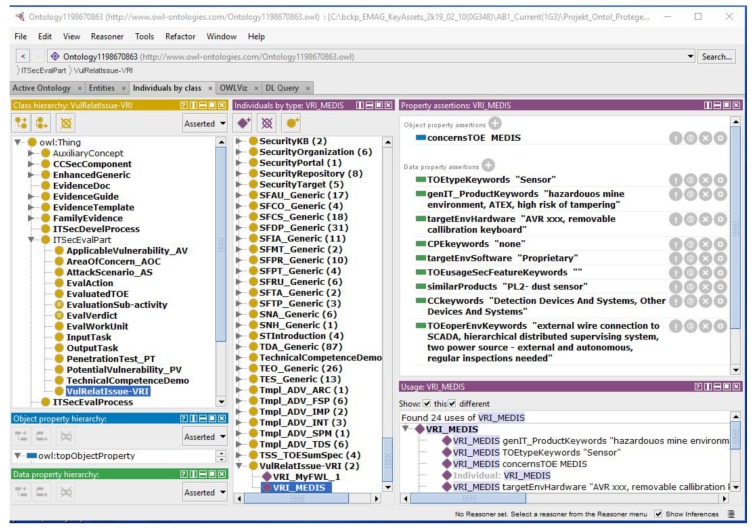
The vulnerability-related issues represented by the ontological model in the Protégé tool.

Figure 2 presents an ontological model example concerning vulnerability-related issues. The left panel shows the class hierarchy of the CCO ontology (CCO-2019-01-07.owl is available on the Supplementary Materials) described in the author’s earlier papers. The *VulRelatIssue_VRI* class has two individuals shown in the middle panel—one for the MyFWL_1 firewall and one for the MEDIS sensor. For the highlighted *VRI_MEDIS* individual, the object and data properties expressing the related knowledge are shown in the right panel. 

Please note that the *VulRelatIssue-VRI* knowledge base expresses “what to search”. The keywords (data properties) used while the sources are searched are:

- TOE-related keywords derived from the security target parts:
  — *TOEtypeKeywords*—defined on a ”TOE type” basis,
  — *TOEusageSecFeatureKeywords*—defined on the basis of an ST section entitled ”usage and major security features of a TOE”,
  — *TOEoperEnvKeywords*—defined on the basis of an ST section entitled ”required non-TOE hardware, software, firmware”,
  — *targetEnvHardware*—hardware platform, e.g., a microcontroller,
  — *targetEnvSoftware*—software platform, e.g., OS,
- keywords implied by the CC product taxonomy,
  — CCkeywords,
- keywords for IT products knowledge bases,
  — *CPEkeywords*—according to [45], if relevant,
  — *genIT_ProductKeywords*—other than those defined by the evaluator, concerning the TOE application, operational environment, specific threats, etc.,
  — *similarProducts*—indicating similar IT products.

The main activities related to Case 2 concern the “EEP2-2 search of public domain sources to identify potential vulnerabilities and attacks” process. The author proposes to develop the *InfoSourceTaxonomy* knowledge base, allowing one to access the knowledge dealing with the potential vulnerabilities and attacks. This way, the evaluators have access to public information sources relevant for the IT products usually evaluated by ITSEF. One of these important sources is the organization responsible for the Common Criteria implementation in the given country and supervising the licensed ITSEFs (scheme owner, certification body). The information source knowledge base defines “where to search” for this knowledge. 

**Figure 3:**
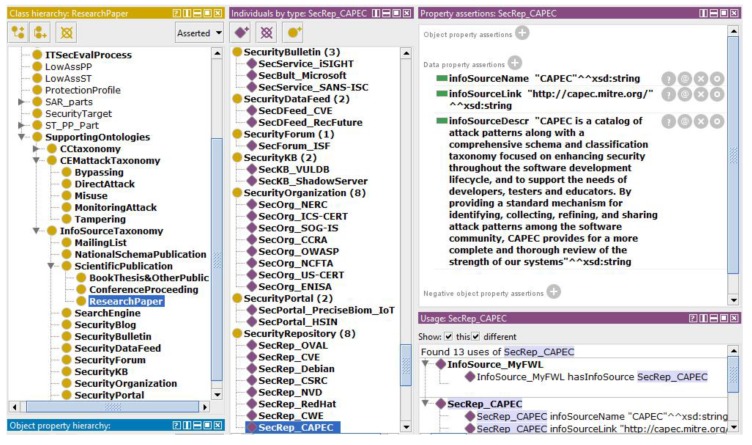
Information sources presented in the Protégé tool.

Figure 3 shows the sources as individuals of ontology classes. The left panel shows the class hierarchy of the CCO ontology, including subclasses of the *InfoSourceTaxonomy* class. The middle panel presents the individuals as examples of sources belonging to different categories. One of them, *SecRep_CAPEC*, is shown in the right panel. Currently, the given source is defined by a short name, description, and link to knowledge in the public service, database, data feed, or internal ITSEF repository. 

The VRI and info sources are the basic input of EEP2-2, but, during the search, the evaluator may look to the evaluation evidences, mainly ADV documents, for details dealing with the TOE design.

EEP2-2 concerns the public domain search and:

1. The evaluator searches the publicly available sources of information (*InfoSourceTaxonomy* knowledge base) to identify possible attack scenarios on the evaluated TOE or similar products, and the vulnerabilities exploited by these attacks.
2. Potential vulnerabilities are registered in the *PotentialVulnerability_PV* knowledge base. The related scenarios are placed into the *AttackScenario_AS* knowledge base.
3. Optionally, only for AVA_VAN.1, if the given potential vulnerability is applicable to the TOE in its operational environment, the analysis should be performed. As the input, the assumptions (a part of ASE_SPD) and security objective for the operational environment (ASE_OBJ) are used.
4. All vulnerabilities considered as “applicable” are marked as the candidates for testing, are placed into the *ApplicableVulnerability_AV* knowledge base, and reported in the ETR (evaluation technical report).
5. The evaluator assigns the verdict (PASS/FAIL/INCONCLUSIVE) for the AVA_VANx.2E and justifies it.

Please note that EEP2-2’s output is placed in three knowledge bases.

The first, *PotentialVulnerability_PV*, includes identified potential vulnerabilities resulting from the search (EEP2-2) or analyses of the vulnerability-relevant items (EEP3-2). The vulnerability specification is based on the main elements of vulnerability description ontology (VDO) [48], expressed by data properties:

- *VulnerabProvenance*—such as the name of the source which provides information [25,42,43,44] and any other source from the *InfoSourceTaxonomy* knowledge base;
- *ExtVulnerabID*—a unique identifier of a vulnerability in an external source, such as a knowledge base article number, patch number, bug tracking database identifier, or a common identifier, such as common vulnerabilities and exposures (CVE) [43] or common weakness enumeration (CWE) [44];
- *VulnerabDescription*—analogous to the VDO ”scenario”;
- *Product*—software and/or hardware configurations that are recognized as vulnerable;
- *AttackTheater*—area or place from which an attack may occur;
- *EngineeringMethod*—method or mechanism used to manipulate the user into interacting with a malicious resource;
- *VulnerabContext*—entity where the impacts are realized from successful exploitation of a security vulnerability;
- *VulnerabilityScore*—according to the common vulnerability scoring system [49];
- *Equipment*—equipment used to exploit a vulnerability;
- *CEMreference*—reference to the CEM attack taxonomy.

Apart from data properties expressing knowledge, the individual includes object properties expressing relations between other individuals belonging to the evaluation project. 

**Figure 4:**
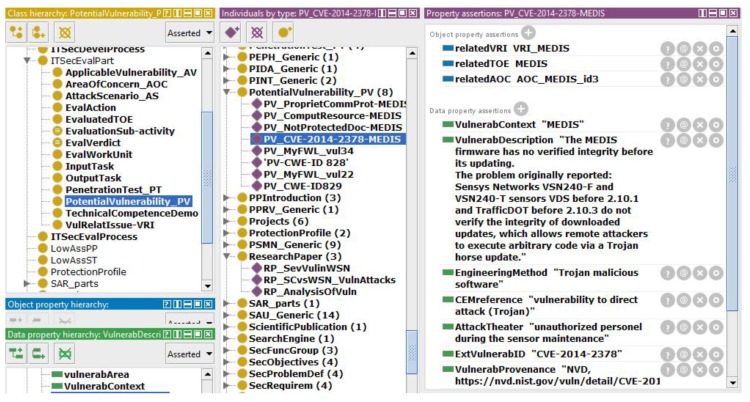
Potential vulnerability presented in the Protégé tool.

The right panel of Figure 4 presents an example of a potential vulnerability specification in the knowledge base. The examples of the considered vulnerabilities are:

- incompatibilities between specific software solutions of sensors and systems of the higher level (host-based) supervising sensors,
- incompatibilities between specific software solutions of sensors and IT cyber security solutions (COTS),
- constrained power and computation resources of sensors faced with time-critical operations implying the use of simplified communication protocols and cryptographic techniques,
- no security domain, no privilege separation,
- vulnerabilities related to wireless devices,
- insecure network architecture,
- enabled trap doors to unauthorized users,
- hardware vulnerabilities [56].

Examples of considered attacks are:

- manipulation of sensor output data,
- Denial of Service—causing system unavailability and breaching time-critical operation,
- forging data designed for the sensor actuator (if any applied in a sensor),
- buffer overflow,
- SQL injection,
- attacks on different layers of the communication stack,
- advanced physical security invasive attacks on hardware, e.g., tampering, probing, voltage/temperature/radiation imprinting, clock glitching, changing environment conditions [56],
- advanced physical security noninvasive attacks on hardware, e.g., simple/differential power analysis (SPA/DPA) or electromagnetic emanations analysis (EMA) [56].

The second EEP2-2 output is placed into the *AttackScenario_AS* knowledge base. The *AttackScenario_AS* ontology class expresses all identified, possible attack scenarios relevant to the given TOE. The individuals of the *PotentialVulnerability_PV* and *AttackScenario_AS* classes are identified concurrently in EEP2-2 and they are closely related, which is expressed by the object properties: *relatedAttackScenario* and *attackDealsWith_PV*.

**Figure 5:**
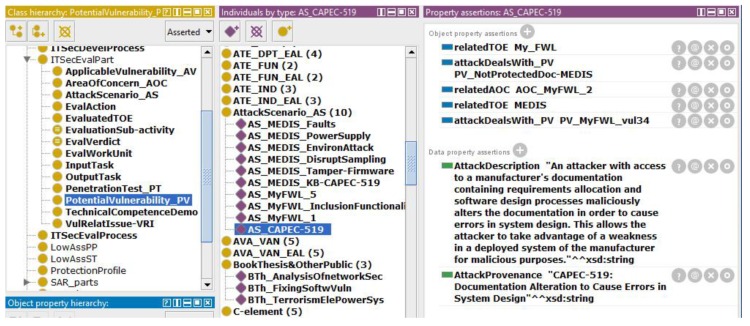
Attack scenario presented in the Protégé tool.

The attack scenarios are expressed by two data properties (Figure 5), extending information included in the potential vulnerability description:

- *AttackDescription*—supplementary description of a scenario,
- *AttackProvenance*—such as the name of the source which provides information.

The third EEP2-2 output related to the ApplicableVulnerability_AV knowledge base concerns only AVA_VAN.1 evaluation. It will be discussed in Case 3, where it is very important.

### 4.3. Case 3 Performing Independent Focused Vulnerability Analysis of the TOE Using the Guidance Documentation, Functional Specification, TOE Design, Security Architecture, Description (and Implementation Representation) to Identify Potential Vulnerabilities in the TOE (AVA_VANx.3E, x = 2,3,4)

For AVA_VAN.2 and higher, the evaluators should identify potential vulnerabilities introduced by the developers. For this reason, evaluation evidences are analyzed with respect to potential vulnerabilities, mainly: ASE (security target), ADV_TDS (design—TOE decomposition to subsystems and modules), ADV_FSP (functional specification—interfaces), ADV_IMP (implementation—source code, electronic schemes), ADV_ARC (architecture—how the architecture is applied to protect security functions), AGD_PRE (preparative user guide), and AGD_OPE (operational user guide). 

Similarly to EEP2-1/EEP2-2, the work embraces two steps:

- planning—deals with “EEP3-1 areas of concern identification”,
- execution—deals with “EEP3-2 vulnerability analysis based on evidences”.

**Figure 6:**
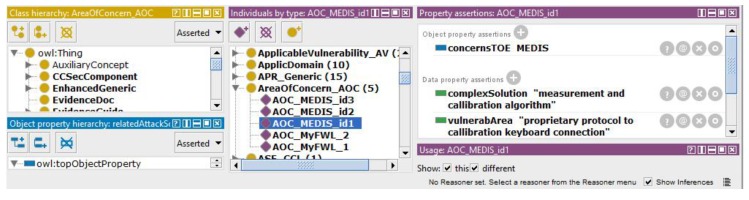
Area of concern representation in the Protégé tool.

As mentioned above, the areas of concern are understood as the suspected, vulnerability-prone parts of the TOE design. Their identification allows orientation of the detailed analysis of evaluation evidences. The *AreaOfConcern_AOC* ontology class is shown in Figure 6. It has two data properties:

- *complexSolution*—e.g., excessively complicated solutions, not well structured areas of the design, areas with too many patches, etc.,
- *vulnerabArea*—e.g., unproven applied technologies, vulnerability-prone technologies, etc.

EEP3-1 concerns the preparation of evidence analysis and involves two activities:

1. The evaluator reviews the TOE evaluation evidences to find different forms of areas of concern, which may include potential vulnerabilities.
2. The evaluator places the identified information in the *AreaOfConcern_AOC* knowledge base. It will be used to orientate the analysis of evidences and, finally, to find potential vulnerabilities.

EEP3-2 is focused on the identification of the second group of vulnerabilities, i.e., evidence-based vulnerabilities, and embraces two steps:

1. The evaluator analyzes the TOE evidences, focusing on the areas of concerns, to identify the potential vulnerabilities and related attack scenarios.
2. The evaluator adds potential vulnerabilities to the *PotentialVulnerability_PV* KB and related attack scenarios to the *AttackScenario_AS* knowledge base.

EEP3-3 analyzes both groups of identified potential vulnerabilities (based on public information searching and evidences analysis) to check whether they are relevant to the TOE’s operational environment (note that for AVA_VAN.1, this work was performed in EEP2-2). Only these relevant, “applicable”, vulnerabilities will be the subject of further analyses. EEP3-3 has three steps:

1. Considering the operational environment description (ASE_SPD and ASE_OBJ), the evaluator analyzes whether each potential vulnerability is applicable to the TOE’s operational environment.
2. All applicable vulnerabilities are marked as candidates for analysis and testing. They are placed into the *ApplicableVulnerability_AV* knowledge base and reported in the ETR.
3. The evaluator assigns the verdict (PASS/FAIL/INCONCLUSIVE) for AVA_VANx.3E (x = 2,3,4) and justifies it.

The *ApplicableVulnerability_AV* knowledge base supplements *PotentialVulnerability_PV* with details (data properties) describing preselected candidates for further analysis and testing:

- *VulCandidateRationale*—why it is considered a candidate for testing;
- *AssumedAP*—implied by the EAL (basic, enhanced basic, moderate, high);
- *CalculatedAP*—calculated attack potential (AP);
- *VulSeverity*—characterizing the vulnerability’s occurrence and severity;
- *VulPriority*—prepares a prioritized list of vulnerabilities for testing;
- *TestReport*—test reports and results (SFRs not met, AP parameters, AP resistance, exploitable or not, etc.);
- *ExploitabilityVerdict*—verdict (not tested, exploitable, not-exploitable, inconclusive, residual).

The contents of the *ApplicableVulnerability_AV* knowledge base are supplemented during further activities. 

For example, the attack potential (AP) can be considered in the next EEP. The EAL claimed for the TOE implies the AP (assumed). The AP is calculated according to the CEM/Annex B methodology [29]. The calculation of the attack potential is focused on the identification of effort required to create the attack which is successfully applied to the TOE. The following factors are assessed in predefined scales and the AP is the sum of these values:

- ET—elapsed time, i.e., time taken to identify and exploit the vulnerability;
- SE—specialist expertise, i.e., the level of generic knowledge of the attacker;
- KT—knowledge of the TOE, i.e., dealing with the TOE design, operation;
- WO—window of opportunity, i.e., considerable amount of access to the TOE or the number of samples of the TOE that the attacker can obtain (related to ET);
- EQ—equipment, i.e., IT hardware/software or other equipment required to identify or exploit the vulnerability.

An example of the software tool calculating the attack potential is presented in a previous paper [57]. 

**Figure 7:**
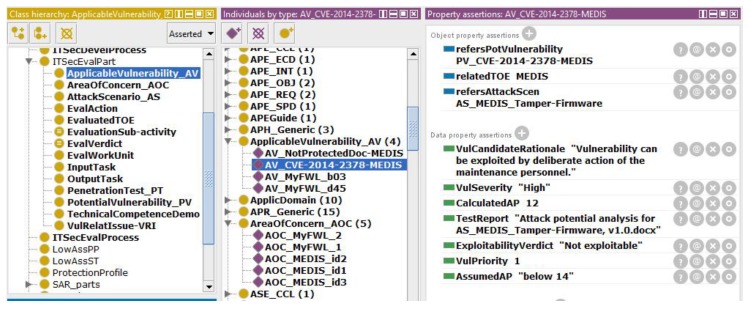
Applicable vulnerability specification in the Protégé tool.

At the end of the AVA_VAN evaluation, the *ApplicableVulnerability_AV* knowledge base includes a complete picture about the relevant TOE vulnerabilities which are summarized in the ETR. Figure 7 presents an example of applicable vulnerability description in the knowledge base. 

Let us consider that for the MEDIS sensor, EAL3 was claimed. This means that MEDIS should be resistant to basic attack (the corresponding AP range is: 10–13). For this reason, *AssumedAP* is “below 14”. The evaluator, considering the relevant vulnerability–attack pair (here: *PV_CVE-2014-2378-MEDIS* and *AS_MEDIS_Tamper-Firmware*), assesses the “attack potential required to exploit scenario”, i.e., *CalculatedAP* = 12. This means that if the minimal AP to exploit a scenario is 12, i.e., “enhanced basic”, then MEDIS is only resistant to attackers with “basic” attack potential. This vulnerability is “not exploitable” for this EAL [29].

### 4.4. Case 4: Conducting Penetration Testing Based on the Identified Potential Vulnerabilities to Determine Whether the TOE is Resistant to Attacks Performed by an Attacker possesing the Given Attack Potential

Case 4 concerns a detailed analysis of candidates for testing (EEP4-1) and penetration tests of these candidates (EEP4-2 to EEP4-5). 

“EEP4-1 Analyzing candidates for testing” supplements information about candidates

1. The evaluator performs an additional search and reviews the evaluation evidences, information sources, guidelines from national schemes, and Common Criteria organizations, e.g., SOG_IS [6,16] to get more information about each candidate.
2. The evaluator calculates the attack potential.
3. The evaluator prioritizes candidates and prepares them for testing.
4. The evaluator updates the *ApplicableVulnerability_AV* knowledge base with this acquired knowledge.

The next elementary processes are focused on devising (EEP4-2), implementing (EEP4-3), and performing penetration tests (EEP4-4). The tests are used to experimentally confirm if the applicable vulnerability is exploitable or not in the TOE’s operational environment. They are iteratively developed and placed in the *PenetrationTest_PT* knowledge base. 

Penetration tests are focused on the evaluated IT product and are performed in ITSEF. Penetration tests have a broad range—from chemical and mechanical experiments, through hardware (SPA, DPA, EMA, environmental, etc.), to network and software experiments typical of IT systems [58]. A test simulates a certain kind of attack related to the considered vulnerability. 

**Figure 8:**
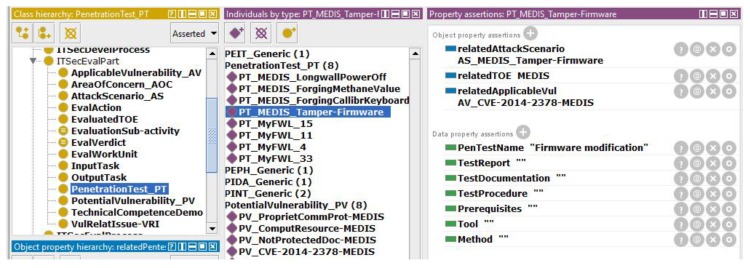
Penetration test specification in the Protégé tool.

A minimal set of object and data properties is proposed for test specification, as shown in Figure 8.

ITSEFs specialize in the evaluation of the selected groups of IT products. The ITSEF profile selection is a business decision and implies an evaluation methodology, personnel competences, and sophisticated software/hardware equipment. The requirements for ITSEF are very high, which influences ITSEF investment and operational costs. 

## 5. Discussion of Validation Results

The above cases involve the whole vulnerability assessment process specified in CEM [29]. The validation was focused on the vulnerability assessment of sensor-related designs and on the possibility of automating this process. 

### 5.1. Case 1 Remarks and Conclusions

EEP1/Case 1 involves typical engineering activities, e.g., verification, configuration, calibration, installation, and cursory testing, based on the delivered evaluation evidences. This task is similar for all kinds of IT products, including sensors.

The planned software tool may support the evaluator in preparing documentation according to CEM.

No knowledge bases are needed for Case 1. 

### 5.2. Case 2 Remarks and Conclusions

The *InfoSourceTaxonomy* knowledge base can be considered a valuable ITSEF asset. It can be reused and extended from one project to another. 

There are many internet services related to IT vulnerabilities and attacks. Most of them are also relevant for sensor networks, e.g., for wireless sensor networks (WSN). 

There are no specialized services for intelligent sensors. Some information can be found on portals of technology providers and organizations dealing with critical infrastructures protection, industrial control systems, and Internet of Things. 

The author proposes developing a repository based on numerous existing publications about sensor vulnerabilities and attacks, e.g., [18,19,20,21]. A systematic and CC-compliant approach to the threat/attack taxonomy was included in the author’s previous paper [37]. The standard ISO/IEC TS 30104 [56] may also be helpful when considering hardware-specific issues. 

The ontological models presented in the paper are very simple. They allow one to create “flat” lists of vulnerabilities and attacks. This is sufficient for a single evaluation project but not to build ITSEF knowledge assets. To build extensive knowledge bases for ITSEF, additional research is needed on taxonomies of vulnerabilities and attacks. 

Particular AVA_VAN components differ with respect to the considered range and details of input information. These differences need further structuring. 

The proposed knowledge bases can be implemented in the software tool supporting the evaluation.

### 5.3. Case 3 Remarks and Conclusions

The rigor of the vulnerability analysis and the considered resistance to attacks performed by an attacker possessing a given potential depend on the claimed EAL:

- AVA_VAN.1 (implied by the EAL1)—no vulnerability analysis, TOE resistance to basic attack;
- AVA_VAN.2 (implied by the EAL2 or EAL3)—independent vulnerability analysis, TOE resistance to basic attack;
- AVA_VAN.3 (implied by the EAL4)—focused vulnerability analysis, TOE resistance to enhanced-basic attack;
- AVA_VAN.4 (implied by the EAL5)—methodical vulnerability analysis, TOE resistance to moderate attack potential;
- AVA_VAN.5 (implied by the EAL6 or EAL7)—advanced methodical vulnerability analysis, TOE resistance to high attack potential (not considered in this paper because no guidance exists; it should be consulted with the “scheme”, i.e., an organization responsible for Common Criteria implementation in the given country).

The current version of the presented methodology (SVAM) does not include details of the vulnerability analysis methodology. General guidance is included in the relevant AVA_VAN work units and in another report [23].

### 5.4. Case 4 Remarks and Conclusions

Penetration tests are generally focused on the security functional requirements (SFR) implemented in the TOE security functions (TSF). They should be harmonized with tests required by the ATE class evidences.

Generally, the evaluator is not expected to provide tests for attack scenarios beyond the resistance level considered for the given AVA_VAN component (EAL).

### 5.5. General Remarks and Conclusions

SVAM is defined as a sequentially ordered set of elementary processes. It can be considered a sequence of activities, but in reality, the work is iterative and more complicated because the evaluators often have to go back to the previously considered activities. This is typical for the CEM methodology. 

The elementary evaluation processes need even deeper structuring allowing one to distinguish details related to the particular AVA_VAN components (activities and data). The EEPs should be refined separately for each component and have implemented work units specific for the EAL (which can be applied when the supporting tool is being developed). The verification of the defined elementary evaluation processes (EEP) versus the contents of the work units defined in CEM was performed previously [51].

The knowledge bases should be ready to acquire knowledge details related to the most rigorous AVA_VAN.4 component. For lower assurance components, certain data properties representing knowledge may not be used or may be formulated less precisely than the high rigor requirements of AVA_VAN.4. This approach may facilitate knowledge reusability from one project to another. When knowledge exists related to the low EAL, it can be reused and supplemented for a higher EAL project. When ITSEF has high EAL knowledge at its disposal, it can also be reused after generalizing the relevant elements.

The *InfoSourceTaxonomy*, *PotentialVulnerability_PV*, *AttackScenario_AS*, and *PenetrationTest_PT* knowledge bases can be the foundation to work out the ITSEF knowledge assets. They have high potential for knowledge reusability and can be maintained in for future evaluation projects. *CEMattackTaxonomy* can be helpful in the organization of these bases.

*CCtaxonomy* has auxiliary meaning and can be used to map the evaluated product to the general taxonomy, and to compare the product with others to find necessary information.

The *VulRelatIssue_VRI*, *AreaOfConcern_AOC*, *ApplicableVulnerability_AV*, and the *PenetrationTest_PT* bases are project-oriented and their reusability is limited.

Currently, a simplified knowledge model is applied. The main concepts are represented by ontology classes. Individuals of these classes have:

- data properties representing knowledge,
- object properties to model relationships between parts of knowledge.

Considering the progress in the development of internet information services, especially data feeds, there is a possibility of deeper knowledge structuring. The planned software tool to support evaluators should be able to acquire external knowledge semiautomatically, but under human or machine learning control.

ETR is a holistic report summarizing the evaluation results of all evidences for the claimed EAL, including the considered AVA_VAN. The CCMODE toolset [7,59] is only designed to support the CC-related development process, but it has potential to be spread to the whole CC methodology as well. The author proposes extending the solutions of the CCMODE project, especially:

- the simplified CEM implementation called “self-assessment”,
- the concept of patterns of evaluation evidences.

The “self-assessment” facility can be extended to full CEM implementation and the set of design patterns can be supplemented by the ETR pattern summarizing all evaluation efforts. The evaluator will be able to generate the ETR report semiautomatically with the use of the tool and on the basis of the ETR pattern and the knowledge acquired during the evaluation. 

The author’s previous papers focusing on IT security evaluation are closely related to the SVAM presented here. A previous paper [53] introduced the general ontological CEM model. This “high-level model” can be used to integrate (within the planned tool) all CEM evaluation processes corresponding to the particular security assurance components embraced by the given EAL. One of the most important processes is the vulnerability assessment process (AVA_VAN) discussed in the paper. Another paper [51] proposed decomposing the CC vulnerability assessment process into “elementary evaluation processes (EEPs)”. There, the EEPs are modelled in a pseudocode and are further developed in this paper with respect to the input and output data models. The main achievements and advancement in the state of the art regarding the author’s previous work are related to the ontology-based I/O data models for EEPs. Another paper [57] presented the implementation of the attack potential (AP) calculator based on the risk analysis tool and scenarios from CEM/Annex B [5]. This calculator can be used in EEP4-1 implementation and the mentioned scenarios use the *PotentialVulnerability_PV*, *AttackScenario_AS* knowledge bases.

## 6. Conclusions

This paper discusses the vulnerability assessment of sensor systems. Sensor systems can be considered an emerging Common Criteria application domain. They belong to the broad IT product family and are used in high-risk applications, yet very few certifications of these products have been completed until now. The sensors’ assurance rises when they are rigorously developed and evaluated by an independent body (ITSEF). The vulnerability assessment is the key issue of the evaluation process, involving different active investigations. Developers and evaluators need dedicated methods and knowledge to support this emerging Common Criteria application domain. The developed SVAM methodology is fully compatible with the Common Criteria evaluation methodology (CEM). 

The vulnerability assessment specified in the Common Criteria methodology can be directly applied to sensors, but it requires refinement related to the structure and the used or produced knowledge. This was a motivation to develop the SVAM method.

The evaluation process may be made more effective by more precisely defining the evaluators’ work (EEPs), facilitating their access to relevant knowledge (ontology, knowledge bases), and automating their work. The structure of the process and data is the first step towards vulnerability assessment automation. Automation can enhance effectiveness and knowledge reusability and decrease the time and cost of the evaluation.

EEPs can be also used as guidelines for evaluators who have hints about the performed activities and necessary data. The data are represented by knowledge bases dedicated to these activities. Please note that ITSEF should have its own CC-compliant, specific evaluation methods for IT product families included in the ITSEF profile. The SVAM can be included in the ITSEF lab developed in the KSO3C project "National schema for the security and privacy evaluation and certification of IT products and systems compliant with Common Criteria". KSO3C aims at establishing a national schema and one of its labs has been established in the author’s organization. This paper’s research results will be used as input for the KSO3C project.

The author plans the following future works:

- modeling of processes and data should be validated on more evaluation projects to achieve higher maturity, allowing one to build a software tool supporting the vulnerability assessment process;
- more validations of SVAM are needed, especially on fully developed evaluation evidences; it is necessary to build more extensive knowledge bases of information sources, potential vulnerabilities, and attacks related to sensor systems;
- extension of taxonomies and knowledge bases of vulnerabilities and attacks;
- refining of ontological models to achieve a higher maturity level,
- structure improvement of the vulnerability analysis methodology (independent, focused, methodical) and added to SVAM;
- integration of an attack potential calculation tool [57];
- embedding the SVAM method into the entire CEM evaluation process.

The author supposes that the deployment of the Cyber Security Act (CSA) [17] will raise interest in sensors systems’ certification and, as a result, the number of certified solutions designed for high-risk applications will increase.

## Supplementary Materials

The following are available online at https://www.mdpi.com/1424-8220/19/11/2518/s1: CCO-2019-01-07.owl: Bialas, A. The Common Criteria Ontology (CCO), 2010–2019.
